# Fusogenic Viruses in Oncolytic Immunotherapy

**DOI:** 10.3390/cancers10070216

**Published:** 2018-06-26

**Authors:** Teresa Krabbe, Jennifer Altomonte

**Affiliations:** 2nd Department of Internal Medicine, Klinikum rechts der Isar, Technical University of Munich, 81675 München, Germany; teresa.krabbe@tum.de

**Keywords:** cancer, immunotherapy, oncolytic, virus, fusion, fusogenic, fusogenicity, immunogenic, syncytium

## Abstract

Oncolytic viruses are under intense development and have earned their place among the novel class of cancer immunotherapeutics that are changing the face of cancer therapy. Their ability to specifically infect and efficiently kill tumor cells, while breaking immune tolerance and mediating immune responses directed against the tumor, make oncolytic viruses highly attractive candidates for immunotherapy. Increasing evidence indicates that a subclass of oncolytic viruses, which encodes for fusion proteins, could outperform non-fusogenic viruses, both in their direct oncolytic potential, as well as their immune-stimulatory properties. Tumor cell infection with these viruses leads to characteristic syncytia formation and cell death due to fusion, as infected cells become fused with neighboring cells, which promotes intratumoral spread of the infection and releases additional immunogenic signals. In this review, we discuss the potential of fusogenic oncolytic viruses as optimal candidates to enhance immunotherapy and initiate broad antitumor responses. We provide an overview of the cytopathic mechanism of syncytia formation through viral-mediated expression of fusion proteins, either endogenous or engineered, and their benefits for cancer therapy. Growing evidence indicates that fusogenicity could be an important feature to consider in the design of optimal oncolytic virus platforms for combinatorial oncolytic immunotherapy.

## 1. Introduction

Cancer immunotherapy represents a promising new aspect of cancer treatment that aims at activating the patient’s own immune system to eradicate the tumor. Ideally, an effective immunotherapy activates the cancer-immunity cycle that starts off with a first round of tumor cell killing, and the activation of the immune system to prime a broad antitumor immune response. This would then induce a second round of tumor cell killing, in which the endogenous immune cells of the patient are activated and directed against specific tumor antigens to eradicate the remaining tumor cells and metastases, as well as provide long-term protection to the patient against recurrence [1].

This process, however, is best achieved by rationally combining different therapeutics [2,3]. While, for example, immune checkpoint inhibitors revolutionized cancer therapy, they only serve to reinforce an already existing antitumor immune response [4]. Oncolytic viruses, on the other hand, can prime the tumor and immune system during the early stages of treatment, thereby mediating optimal outcomes in response to subsequent immunotherapeutic approaches. This strategy demonstrated significant effects in clinical trials using oncolytic viruses to prime solid tumors for immune checkpoint inhibition [5,6,7]. Oncolytic viruses (OVs) are viruses that have an intrinsic or engineered mechanism for tumor-specific replication and subsequent cell killing. OVs exert their effects both via the direct killing of infected tumor cells, as well as via indirect effects, such as destruction of tumor vasculature, and induction of adaptive immune responses, which can be directed against the tumor and lead to the destruction of neighboring uninfected tumor cells. Furthermore, the evolution of virus engineering methods allows us to design and rescue recombinant viral vectors from plasmid DNA. In this way, viruses can be modified to increase tumor specificity or to express therapeutic genes and/or reporter genes. Over the last decade, significant progress was made in the development of enhanced OV therapies [8,9], and a variety of vectors entered clinical trials [10,11,12]. Increasingly, the use of naturally occurring fusogenic OVs, or recombinant vectors engineered to express fusion proteins, is becoming a provocative strategy for enhanced oncolytic effects.

Fusion is a common cellular process that enveloped viruses utilize to mediate the merging of the viral envelope with the host membrane during infection and internalization as a critical first step in their virus life cycle. Virus–cell fusion is achieved by one or more viral surface glycoproteins, denoted as fusogenic membrane glycoproteins (FMGs) or simply fusion proteins, which interact with receptors and coreceptors on target membranes, and induce distinct fusion processes according to their protein structure [13]. In addition to their function for virus entry into the host cell, certain virus fusion proteins also induce cell–cell fusion when expressed on the cell surface of an infected cell, thereby mediating viral spread and virulence [13,14]. Cells infected with these viruses form areas of non-viable, multinucleated giant cells, so-called syncytia, as the viral-expressed fusion protein is shuttled to the cellular membrane surface, where it mediates fusion of the infected cell to neighboring uninfected cells [15]. Because of the dual role of viral fusion proteins in cell entry and viral spread via syncytia formation, they are becoming increasingly attractive in the field of oncolytic virus development, as they offer a unique and efficient mechanism of tumor cell killing through fusion of tumor cells, and via potent induction of immune responses. In this review, we provide an overview of the mechanisms of virus-mediated cellular fusion, as well as a summary of naturally occurring fusogenic viruses and oncolytic viruses that are engineered to exploit the benefits of heterologous viral fusion proteins for cancer therapy. We also discuss the status of fusogenic oncolytic viruses in clinical translation.

### 1.1. OV-Mediated Fusion as an Oncolytic Strategy

Oncolytic viruses with fusogenic glycoproteins have some desirable advantages over their non-fusogenic counterparts, influencing both direct oncolytic and immune-stimulatory effects, which may translate to therapeutic benefits upon clinical translation. Tumor cells infected with these viruses form syncytia. As infected cells fuse with neighboring cells, intratumoral spread of the infection is facilitated [15]. Another potential advantage of this mechanism of spread through a tumor is the minimal release of mature virions into the surrounding healthy tissue or into systemic circulation. Moreover, the ability to cause infected cells to fuse with neighboring tumor cells allows single virions to potentially result in the death of large numbers of tumor cells as they are pulled into the growing syncytium [16]. This implies that these viruses can efficiently destroy the tumor without producing high titers of virus, which is beneficial in contributing to a safe therapeutic agent with a wide therapeutic index. This mechanism can also allow for additional rounds of replication without exposure to neutralizing antibodies. 

**Figure 1:**
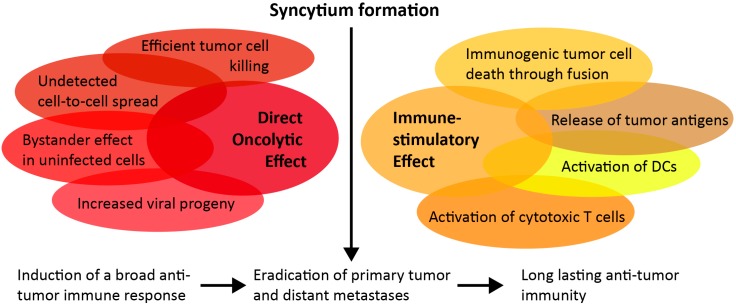
Advantages of syncytia formation for immunotherapeutic approaches. Upon tumor cell infection with a fusogenic oncolytic virus, syncytia form and contribute to an enhanced direct oncolytic effect, resulting in wider spread of the infection and an increased release of viral progeny from a large multinucleated area. Syncytia formation also prompts a broad immune-stimulatory effect. Together with immune-activating cytokines, tumor associated antigens (TAAs) are released from dying syncytia, and are immediately taken up by recruited antigen-presenting cells (APCs) that prime a cytotoxic T-cell response by cross-presentation through dendritic cells (DCs).

The syncytia are viable for only a short time, before they undergo immunogenic cell death (ICD) [17]. Fusogenic viruses mediate potent antitumor immune responses by acting as immune-adjuvants, as exposed viral antigens and products from dying cells provide danger signals, such as damage-associated molecular patterns (DAMPs) and pathogen-associated molecular patterns (PAMPs) to activate immune cells [18]. Typical markers of ICD include cell surface exposure of calreticulin (ecto-CRT) and the release of heat-shock proteins (HSP70 and HSP90), high-mobility group box 1 (HMGB1), ATP, and uric acid from dying cells [19]. In response to this ICD, cytokines are released, and the immune suppressive tumor microenvironment is modulated [20,21,22]. Due to the unique mechanism of cell death through fusion, syncytia formation is especially immunogenic, and releases a broad range of tumor antigens and promotes their cross-presentation by dendritic cells to cytotoxic T cells [23,24]. Furthermore, the death of syncytia, induced by fusogenic viruses, is associated with autophagy, which enhances tumor immunogenicity [23,25]. By priming a strong and durable antitumor immune response, fusogenic viruses could confer long-lasting immunity. A summary of the antitumoral mechanisms of OV-mediated fusion is depicted in Figure 1.

### 1.2. Mechanisms of Virus-Mediated Fusion

Over the last two decades, major breakthroughs were made in the understanding of the proteins and protein complexes that are responsible for mediating fusion between viral envelopes and their host cells. It is now clear that all characterized fusion proteins are similar, in that they mediate membrane fusion through irreversible conformational changes involving a trimer-of-hairpins motif, as well as through the induction of a common pathway of membrane dynamics to coerce membranes to join together via lipid junctions [13,26]. Membrane fusion requires that membrane bilayers come into intimate contact, facilitating a key step called hemifusion, in which small regions of the outer monolayers merge, before the two bilayers form a fusion pore, which then expands via a high-energy reaction until the fusion is complete and the membranes become one. This mechanism of membrane fusion is also employed during cell–cell fusion reactions. The conformational changes in transmembrane fusion proteins occur in response to specific triggering mechanisms, such as binding to cellular receptors and/or a drop in pH. Fusion can, therefore, occur at the cell membrane or within the acidic endosomal compartment. These conformational changes result in the exposure of hydrophobic fusion peptides (FPs) or fusion loops (FLs), which interact with cell membranes, causing destabilization of the membrane and subsequent fusion [13]. The fusion protein exists on the packaged virus envelope in its native state, which is often metastable. The membrane-embedded prehairpin intermediate occurs as a homotrimer of the fusion peptide before conversion to a compact trimer-of-hairpins structure, which is generally the most energetically stable conformation [26]. Despite the ubiquity of the fusion process, the viral fusion proteins themselves are quite diverse in their sequences, structures, and triggering mechanisms. For a comprehensive review of the diverse viral fusion proteins, a variety of publications can be consulted [13,26,27]. Interestingly, even the non-enveloped small reoviridae family developed fusion-associated small transmembrane (FAST) proteins, suggesting an evolutionary advantage of viruses capable of fusogenicity, even for naked viruses [28]. In contrast to the fusion proteins encoded by enveloped viruses, FAST proteins predominantly mediate cell–cell fusion rather than virus–cell fusion [29,30]. 

## 2. Cytopathic Effect of Fusogenic Proteins

Although the predominant function of fusogenic membrane glycoproteins is to mediate the process of infection via fusion of the virus envelope with the target cell, a side product of this is the induction of fusion between infected and non-infected cells. Viral glycoproteins are shuttled to the membrane surface of infected cells, and interact with their receptors on neighboring cells. A sequence of conformational changes prompts the fusion of these cells, and over time, causes the formation of merged multinucleated giant cells [13]. These syncytia can be viable for several days before losing their cellular membrane integrity and, consequently, their viability. During this time, they continue to fuse with neighboring cells to form large syncytial areas of up to 100 nuclei/syncytia [17]. The cytopathic processes of fusogenic proteins and syncytia formation, although investigated, are not completely resolved; however, syncytial death is associated with nuclear fusion, premature condensation of chromosomes, severe ATP depletion, and autophagic degeneration [23]. This process is also accompanied by the release of exosome-like vesicles, termed syncytiosomes. Factors, such as the viral backbone and properties of the infected tumor cell type, seem to influence not only the kinetics of syncytia formation, but also the type of cell death the syncytia eventually undergo. 

Apoptosis of syncytia was noted in cells infected with human immunodeficiency virus (HIV)-1 [31,32]. Apoptotic cell death observed in cultures of HIV and other syncytium-forming viruses is primarily due to the amplification of background apoptosis in the wake of cell-to-cell fusion [33]. In measles virus-induced syncytia, DNA fragmentation within the nucleus indicative of apoptosis was demonstrated by flow cytometry, agarose gel electrophoresis, and electron microscopy [34]. The reovirus FAST proteins lead to apoptosis-induced membrane instability [15]. The apoptotic pathway is also activated in glioma cells lines, upon transfection with the fusion proteins of the gibbon ape leukemia virus (GALV) and measles virus (MV-F/HN) [35]. Apart from apoptosis, the necrotic pathway is also induced under some conditions. It was demonstrated that transfection of Hep3B cells with GALV leads to mitochondrial dysfunction prior to loss of viability, and this mechanism of cell death cannot be inhibited by the pan-caspase inhibitor, carbobenzoxy-valyl-alanyl-aspartyl-(*O*-methyl)-fluoromethylketone (Z-VAD-FMK) [17]. This holds true for transfection of non-small-cell lung cancer cells (NSCLCs) with the different fusion proteins from human endogenous retrovirus type W (HERV-W) or feline endogenous virus (RD-114), which led to the conclusion that non-apoptotic processes must be involved [36]. Furthermore, transfection of the human leukemia cell line, HL-60, with GALV led to the overexpression of HSP70, which inhibited the nuclear translocation of p65. The cell-killing effect of fusion was partially mediated by its inhibitory effect on nuclear factor kappa B (NF-κB) [37]. 

An important feature of syncytial cell death is a significant bystander effect on neighboring, uninfected cells. “Contagious apoptosis” was displayed by HIV-infected cluster-of-differentiation-4 positive (CD4^+^) cells upon fusion of dying cells with neighboring, “healthy” cells [31]. In measles virus-infected cells, where DNA strand breaks were visualized by end labeling with terminal transferase, central nuclei showed staining, while nuclei at the periphery of the giant cell did not, suggesting that cells which have not begun the process of DNA fragmentation (a relatively early step in apoptotic cell death), and possibly even cells which are not infected, are being actively recruited into these multinucleated giant cells [34]. This bystander effect also increases the spread and the area that produces virus progeny, leading to better replication kinetics [38].

With cancer immunotherapy in mind, it is important to consider the immunogenicity of a therapeutic approach. Since the general activation of an immune response against antigens associated with dead cells is suppressed to ensure whole-body homeostasis, this mechanism has to be turned around to produce a systemic immune response against tumor cells. Immunogenicity of cell death is dependent on a combination of antigenicity (i.e., the release of neo-epitopes) and adjuvanticity (i.e., activation of specific DAMPs) [20]. Although it is generally recognized that all oncolytic viruses can induce an immunogenic cell death (ICD) [39,40], growing evidence indicates that tumor cell death via syncytia formation is particularly immunogenic, and could represent an important mechanism involved in the antitumor efficacy of fusogenic viruses [41,42,43]. Morphological and biochemical evidence was found for apoptotic, as well as necrotic and autophagic, cell death associated with syncytia formation. This underlines the notion that the strict division between apoptotic, non-immunogenic, and necrotic, immunogenic cell death is misleading, and that cell death through fusion is a very heterogeneous process depending on various, and not completely elucidated variables [44]. 

Immunogenic cell death is associated with the release of danger signals such as heat-shock proteins, which stimulate the uptake of dead cell-associated antigens [20,45]. A release of HSP70 was confirmed from different tumor cells transfected with GALV, as well as the release of gp69, another indicator of immunogenicity [46]. To gain a deeper understanding and to demonstrate immunogenic cell death of dying syncytial cells, Bateman et al. injected immune-competent mice with GALV-transfected, syngeneic syncytial tumor cells or tumor cells transfected with a control plasmid to elicit an immune response. They showed slower tumor growth and protection of vaccinated animals against rechallenge with the fused tumor cells. They associated this phenomenon with the release of more syncytiosomes from GALV-induced syncytia than the exosomes released from cells dying via other mechanisms. These syncytiosomes load dendritic cells more efficiently to prime an antitumor immune response [23]. FMGs also reverse the inhibitory effects of tumor cells on DCs to potentiate interleukin (IL)-12 production, and naive T-cell priming [24]. Additionally, they increase the effective cross-presentation induced by syncytiosomes significantly compared to that of cells dying through herpes simplex virus thymidine kinase (HSV-1 TK)/ganciclovir-killed tumor cells [23]. FMGs also have a higher bystander effect than that produced by suicide genes, such as HSV-1 TK [35,46].

Apart from dead tumor cells, the virus infection itself is detected by Toll-like receptors (TLRs), cytosolic DNA sensors, retinoic acid inducible gene (RIG)-like receptors or nucleotide-binding oligomerization domain (NOD)-like receptors, depending on the virus. This causes the release of intracellular danger signals, which alert the innate and adaptive immune system. This anti-viral response may also be able to activate an antitumor immunity relying on cross-presentation of tumor antigens from infected cells on antigen-presenting cells (APCs). Virus–cell fusion is sensed by the innate immune system, and activates a stimulator of interferon genes (STING)-dependent signaling pathway that leads to the production of type I interferon (IFN) and molecules encoded by IFN-stimulated genes (ISGs). A similar response occurred following cell–cell membrane fusion [47]. For the measles virus, it was demonstrated that its replication triggers a basal IFN-β response independently of hemagglutinin (H) and fusion (F) proteins, but cell–cell fusion amplifies this response [22]. These viral immunogens act as potent adjuvants, and in combination with fusion-mediated cell death, may provide an optimal pathway to generate a broad antitumor immune response [48].

## 3. Fusogenic Oncolytic Viruses and Their Potential in the Clinic

### 3.1. Viruses with Endogenous Fusion Proteins

Perhaps due to the evolutionary advantage associated with the ability to induce fusion, many different virus families adopted this mechanism of host cell entry and/or spread. Keeping in line with the focus of this review, we only discuss those fusogenic viruses that are oncolytic.

Although all enveloped viruses enter the cytoplasm via membrane fusion, the mechanism can vary depending on the virus [14,49]. The envelopes of some viruses, such as paramyxoviruses, poxviruses, and herpes viruses, fuse directly with the cell membrane after binding to the host cell receptor. Other enveloped viruses, such as the rhabdoviruses, enter their host cells via endocytosis, but fuse with the endosome to release their genomes into the cytoplasm upon acidification within the endosomal compartment [50]. Because these viruses rely on a reduced pH to trigger fusion, they do not cause fusion at the cell plasma membrane, and therefore, do not typically induce cell–cell fusion.

Of the paramyxoviruses, the measles virus (MV) and Newcastle disease virus (NDV) were extensively applied as oncolytic virus therapeutics, and to a lesser extent, the mumps (MuV) and Sendai virus (SeV) also show promise. These viruses initiate infection via attachment to their cellular receptors, allowing fusion of the viral envelope to the host cell membrane. This process is coordinated by the activities of two discrete transmembrane glycoproteins: an attachment protein and a fusion (F) protein. The receptor for most paramyxoviruses is a molecule containing sialic acid residues, although it was demonstrated that MV has at least 3 different receptors: the complement regulatory molecule, CD46, the signaling lymphocyte-activation molecule (SLAM, CD150), and the cell adhesion molecule, Nectin-4 [51]. The virus attachment proteins are named hemagglutinin-neuraminidase (HN), hemagglutinin (H), or glycoprotein (G), depending on the virus.

Although the F protein directly mediates membrane fusion, paramyxoviruses require the co-expression of the attachment protein in order for fusion to occur [52]. This event is also pH-independent, meaning it does not require acidic conditions for activation of fusion, allowing the initiation of infection to occur at the plasma membrane of the host cell. Due to this independence of low pH, also infected cells expressing viral glycoproteins on their surface can fuse with adjacent cells, resulting in syncytia formation [53]. Typical paramyxovirus F proteins are synthesized as a precursor, F_0_, which must be proteolytically cleaved to form F_1_ and F_2_ for fusion activity. These F_1_ and F_2_ polypeptides are disulfide-linked, and are derived from the carboxyl- and amino-terminal domains, respectively [54]. The sequence of the cleavage site is an important determinant of the cellular site of cleavage. F proteins that have a furin recognition site are cleaved in the trans-Golgi domains, and therefore, are delivered to the plasma membrane in their active form. In contrast, F proteins that have single basic residues at the cleavage site are delivered to the plasma membrane in an inactive, uncleaved form, and require extracellular host cell enzymes in order to direct membrane fusion. Although all paramyxovirus F proteins are glycosylated, the location and number of carbohydrate addition sites are not at all conserved [55]. 

As negative-strand RNA viruses, paramyxoviruses are particularly sensitive to the anti-viral actions of type I IFNs. Cancer cells acquire various mutations along the IFN signaling pathway during their malignant transformation, which help them to escape from host regulation [56,57], and also conveniently provide oncolytic viruses with the opportunity to replicate and destroy them in the absence of a productive anti-viral response. This is, in large part, the mechanism by which oncolytic viruses obtain their tumor specificity [58]. The overexpression of viral receptors on the surface of cancer cells may also contribute to the specificity of some paramyxoviruses for tumor cells. Specifically, the abundant expression of sialoglycoproteins on the surface of cancer cells [59] likely enhances the association of NDV, MuV, and SeV for which sialic acid-containing sialoglycoproteins are the cellular receptors [60,61].

In addition to the potential tumor-targeting advantage of using sialic acid residues as receptors for paramyxovirus attachment, the sialidase (neuraminidase) activity of the HN protein of SeV, NDV, and other paramyxoviruses provides a unique potential advantage through the ability to remove sialic acid residues from the surface of tumor cells [43,62,63]. The relatively high density of sialic acid glycoproteins on tumor cells is thought to increase the invasive potential of the tumor by creating a “coating” on the cell surface that serves to hide tumor antigens, and provide a mechanism of escape from immune surveillance. Furthermore, the overexpression of sialic acid creates a net negative charge on the cell surface, leading to repulsion of cells and facilitating cancer cell entry into the blood stream, and the metastatic potential of tumor cells seems to correlate with the abundance of sialic acid residues for a variety of malignancies [64,65]. Therefore, the removal of these sialic acid residues from the surface of malignant cells via viral-mediated sialidase activity can unmask some of the tumor antigens, and render the cells visible to the immune system. Indeed, the removal of sialic acids from tumor cells is associated with the inhibition of tumor growth, the activation of natural killer (NK) cells, and the secretion of IFN-γ [66]. Neuraminidase activity can cleave and remove sialic acid residues from malignant cells, resulting in substantially enhanced induction of T-cell responses [67].

Due to the many attractive properties of paramyxoviruses as oncolytic agents, several viruses from this family were investigated for their potential as cancer therapeutics. Sendai virus, although a highly transmissible respiratory virus in rodents, is considered apathogenic in humans. Preclinical studies using recombinant SeV (rSeV) demonstrate that the virus can spread extensively in tumor xenografts, leading to tumor growth inhibition for a wide variety of tumor entities, while leaving healthy surrounding cells unharmed [68,69]. These results were also reproduced in rat models of melanoma, neuroblastoma, hepatocellular carcinoma, squamous cell carcinoma, and prostate cancer, where it was demonstrated that rSeV could be a potent immune booster for DC-based cancer immunotherapy [70]. Interestingly, the replication of SeV does not seem to be a requisite for a therapeutic effect, as even UV-inactivated SeV was shown to provide efficacy in a variety of tumors [71,72]. These antitumor effects were further enhanced through the conjugation of IL-12 with HN-depleted viral particles, which resulted in a novel immune-stimulatory pseudovirion, which suppressed metastatic melanoma growth through enhanced IFN-γ production [73].

NDV is an avian virus that is extensively studied for its oncolytic potential. Depending on their virulence in their avian hosts, NDV strains are classified into three categories: velogenic (highly pathogenic), mesogenic (moderately pathogenic), and lentogenic (mildly pathogenic). This classification is based on the resultant mean death time in embryonated chicken eggs [74]. The virulence of NDV strains is determined by the cleavage site of the fusion protein, the stem region and globular head of the HN protein [75,76], and the accessory protein, V, which functions as an interferon antagonist [77]. Mesogenic and lentogenic strains of NDV were tested in a wide range of human and rodent tumor cells in vitro [76,78,79], as well as in preclinical rodent tumor models in vivo [80,81,82]. Interestingly, lysates from NDV-infected tumor cells could also be used to pulse dendritic cells, resulting in potent T-cell responses compared to DCs pulsed with uninfected tumor lysates [83]. The use of reverse genetics to manipulate the genome of NDV vectors further enhanced the flexibility and efficacy of NDV as an oncolytic agent. A modification to the F protein of the strain Hitchner B1 to introduce a multibasic cleavage and activation site (rNDV/F3aa), which allows for induction of fusion activity in the absence of exogenous proteases, resulted in enhanced tumor cell killing through induction of large intratumoral syncytia [84]. We demonstrated that further modification of this vector to introduce a single amino acid substitution (leucine to alanine at position 298, L289A) in the F protein results in substantially augmented fusogenicity, which is also active in the absence of the HN protein, and causes efficient tumor-specific syncytia formation in vitro and in vivo for orthotopic hepatocellular carcinoma in rats [85]. However, despite the promising preclinical, as well as clinical, data to support these mesogenic strains of NDV as potent oncolytic agents, the further development of these vectors was substantially hampered by the classification of these strains as select agents by the United States Food and Drug Administration (USFDA) in 2008 [86].

Although a human pathogen, the measles virus (MV) was explored for its potential as an oncolytic virus for over a decade, and evidence for its anticancer effects, both preclinically and clinically, is growing [87,88,89]. Particularly, the Edmonston B vaccine strain was shown to have a favorable safety profile and promising oncolytic effects. Recombinant MV-based vectors with enhanced oncolytic potential are also under development. One important strategy is to retarget the virus to alternate receptors to reduce off-target effects owing to the widespread distribution of its native receptors. This can be accomplished by elongating the viral attachment H-glycoprotein with an ankyrin repeat protein [90]. Another strategy is to create a pseudoreceptor system using single-chain antibody fragments to retarget the virus to tumor-specific CD38, epidermal growth factor receptor (EGFR), or EGFR mutant vIII (EGFRvIII) [91]. Similarly, another strategy involves engineering of a fully retargeted MV recombinant displaying tumor-specific receptor binding ligands in combination with mutations to ablate attachment via CD46 and SLAM receptors [92]. In efforts to facilitate in vivo monitoring of viral replication, MV constructs expressing carcinoembryonic antigen (MV-CEA) and the sodium-iodine symporter (MV-NIS) were constructed [93,94]. To further enhance the therapeutic potential of MV, a new recombinant was engineered to express the prodrug-converting enzyme for 5-FC, which converts the nontoxic compound into a highly cytotoxic drug. In vivo investigations demonstrated that intratumoral application of the suicide gene-expressing MV in combination with systemic 5-FC therapy resulted in a significantly enhanced reduction of tumor burden when compared with virus treatment only in a xenograft mouse model [95]. Despite promising preclinical data, the clinical translation of oncolytic MV has been slow and is yet to progress beyond early-phase clinical trials.

The mumps virus (MuV), although explored to a much lesser extent than the other oncolytic paramyxoviruses in the preclinical setting, was applied in clinical investigations for various human cancers more than forty years ago in Japan with promising outcomes [96,97,98]. The Urabe strain was recently obtained and used as the basis for reverse genetics to generate recombinant vectors for further development of MuV as a therapeutic agent [99]. An important consideration, however, in the utilization of human viruses, such as MV and MuV, as therapeutic agents, is that the general population is vaccinated against these viruses. It is speculated that the presence of circulating neutralizing antibodies, as a result of vaccination, could interfere with the efficacy of these viruses when applied as cancer therapeutics. Whether or not this is actually the case remains to be fully elucidated.

### 3.2. Viruses with Engineered Fusion Proteins

One of the greatest advantages of oncolytic viruses is the possibility to engineer individualized vectors depending on the patient’s needs. This may include introducing additional safety features, improving efficacy of tumor cell killing, or adding any other kind of therapeutic gene. Since the early 2000s, various groups demonstrated that oncolytic virotherapy could be improved by introducing fusogenic proteins (see Table 1).

With these engineered viruses, the advantages of fusion for viral replication and spread become evident in direct comparison with their non-fusogenic counterparts. Since syncytia are viable for at least 24 h after formation, this form of cellular adaptation permits the intratumoral spread and release of viral progeny within the confines of the tumor. Extensive syncytium formation mediated by the reovirus FAST proteins triggers localized cell-to-cell transmission of the infection, followed by enhanced progeny virus release from apoptotic syncytia [15]. Moreover, a conditionally replicative adenovirus equipped with human immunodeficiency virus type 1 (HIV-1) envelope glycoproteins was able to increase spread and facilitate virion release from syncytia-forming CD4^+^ cells when compared with non-syncytial cells [100]. The fusogenic GALV protein was shown to enhance intratumoral spread and antitumor activity when introduced into an adenoviral vector [38]. Engineered viruses may even surpass viruses with endogenous fusion proteins in their fusogenic abilities. A recombinant vesicular stomatitis virus vector (VSV-FH), in which the endogenous VSV glycoprotein G was replaced with MV-F and MV-H glycoproteins, yields more viral progeny, and presents faster replication kinetics and larger fusogenic capabilities than measles virus. This effect can be attributed to the fact that viral RNA and proteins are produced faster and in higher quantities in VSV-FH-infected cells, due to the rapid life cycle of the VSV vector [101]. In a similar strategy, an optimized F protein from NDV (NDV/F3aa(L289A)) was expressed in a recombinant VSV vector (rVSV-F(L289A)) in order to introduce fusogenicity to the vector platform. This modified vector resulted in efficient syncytia formation and a significant survival advantage in tumor-bearing rats over the parental VSV vector in an orthotopic hepatocellular carcinoma model in immune-competent rats [102,103].

The effects of fusogenic oncolytic viruses in vivo are important for predicting their potential value in clinical translation. A replication-competent adenovirus expressing GALV (ICOVIR16) was tested in different tumor models, and showed enhanced antitumor activity in subcutaneous SkMel-28 and NP18 lesions after both intratumoral and systemic administration compared to its non-fusogenic counterpart (ICOVIR15) [38]. The antitumor effects of the HSV-1+ GALV + Fcy::Fur (OncoVEX^GALV/CD^) were tested in various cell lines in vitro and tumor lesions in vivo (see Table 1). A striking finding was that, at a 5-fold lower dose, OncoVEX^GALV^ retains its ability to cause tumor shrinkage in subcutaneous lesions of human squamous cell carcinomas (Fadu) or human fibrosarcomas (HT1080) in the flank of BALB/c homozygous nude (nu/nu) mice, whereas non-fusogenic OncoVEX does not [107]. VSVΔM51 expressing the p14 FAST protein demonstrated increased oncolytic activity against MCF-7 and 4T1 breast cancer spheroids in culture and survival prolongation in a primary 4T1 and a CT26 metastatic colon cancer model in vivo. Increased numbers of activated CD4^+^ and CD8^+^ cells were also detected in tumors [104]. Additionally, in lung metastases of human prostate cancer xenografts, intravenous administration of an HSV vector engineered to express GALV (Synco-2D) produced a significant reduction of tumor nodules by day 40 post-inoculation [106]. Preclinical results suggest that fusogenic oncolytic viruses have the potential to outperform their non-fusogenic counterparts in prolonging survival and inducing an antitumor immune response. They also illustrate that various tumor types can be targeted; however, additional studies are needed to investigate which virus works best with which fusion protein in which tumor.

### 3.3. Clinical Trials

Until now, viruses engineered to express heterologous fusion proteins were not yet applied to clinical studies; however, the application of naturally fusogenic oncolytic viruses, namely the paramyxoviruses, was extensively explored in clinical studies. Although we only provide a brief summary of clinical trials of oncolytic viruses that induce cell–cell fusion, a more comprehensive review can be obtained elsewhere [62,115,116].

Administration of oncolytic NDV vectors via various routes of administration up to relatively high doses resulted in mild-to-moderate adverse effects, such as mild conjunctivitis, laryngitis, and flu-like symptoms in clinical investigations [116]. NDV strains applied in clinical trials include MTH-68/H, NDV-PV701, NDV-Ulster, and NDV-HUJ. These studies investigated both the direct oncolytic application of the virus [117,118,119,120,121], as well as combinations with oncolysates and whole-cell vaccines prepared from cancer cells by autologous or allogenic transfer [122,123,124]. A phase III trial involving 50 colorectal carcinoma patients, treated by immunization with an NDV-infected autologous tumor cell vaccine following resection of hepatic metastases, unfortunately revealed no significant difference in survival in treated and control patients; however, interestingly, an increase in the 10-year overall survival was observed in the subgroup of colon cancer patients treated with the therapy [125].

Along with NDV, MV was also investigated in numerous phase I and II clinical trials. These trials all involve attenuated MV-Edmonston, and either the unmodified Edmonston Zagreb (MV-Ez) strain or engineered vectors expressing the NIS or CEA reporter genes. The earliest reported clinical trial involving MV was a phase I investigation of MV-Ez injected intratumorally in patients with cutaneous T cell lymphoma [126]. Impressively, tumor regressions occurred in three out of five patients, and regression of distant uninjected lesions was also observed, even in the presence of preexisting MV antibodies [126]. Following this initial success, the Mayo Clinic in Rochester, Minnesota is the leading player in clinical investigations involving oncolytic MV. Phase I trials employing recombinant MV-NIS and MV-CEA were conducted in ovarian cancer [93,127] and multiple myeloma patients [128]. In these studies, no dose-limiting toxicities were observed, and fever, abdominal pain, and fatigue were the most commonly reported side effects. There was no evidence of viral shedding in saliva or urine [127]. The outcomes of these trials are encouraging, with numerous patients demonstrating complete or partial tumor regression or stable disease. In a phase I trial using MV-NIS in patients with refractory multiple myeloma, the first treated patient exhibited a complete response that was durable for nine months after receiving a single high dose of virus (10^11^ TCID_50_) [128].

As previously mentioned, mumps virus was utilized during the 1970s in several clinical trials in Japan, with both wild-type and attenuated vectors in patients with advanced malignancies [96,97,129]. A majority of patients in these studies experienced long-term regression or control of tumors, as well as a reduction of ascites, edema, and cancerous bleeding, although a long-term survival benefit was not reported [62]. UV-inactivated Sendai virus was recently applied in a phase I/IIa study in patients with advanced malignant melanoma at Osaka University in Japan [130]. Live versions of the virus were also applied in small case studies in patients with advanced cancers, which were well tolerated and resulted in impressive response rates [62].

## 4. Conclusions and Outlook

Extensive preclinical and clinical research in the field of oncolytic virotherapy, spanning the course of more than a century, recently culminated in groundbreaking advancements. The concept of using viruses as therapeutic agents transcended science fiction thriller plots to becoming a clinical reality in the management of cancer patients. Although oncolytic viruses are now generally accepted as promising cancer treatment agents, there remains a huge logistical challenge to make a comprehensive comparison of the myriad oncolytic virus vectors under development, in order to determine which are the most suitable for clinical translation. Furthermore, it is highly questionable that there will ever exist a “one-size-fits-all” virus that is effective in all cancer entities and patient settings. However, despite these points, both basic scientists and clinicians alike would agree that certain features are ubiquitous in the conceptualization of an ideal vector platform. The essential aspects include the following:

1. Little or no toxicity at effective doses;
2. Tumor-selective replication;
3. Rapid spread throughout the tumor mass, resulting in efficient tumor debulking;
4. The ability to induce potent adaptive antitumor immune responses.

We believe that the abundant research reviewed here would argue that oncolytic viruses that encode for a fusogenic protein, whether it be endogenously expressed or introduced via viral engineering, represent optimal OV platforms that fulfill all of the desirable requisites. Due to the widespread applicability of virus engineering and reverse genetics rescue systems, heterologous fusion proteins can be engineered into a variety of oncolytic platforms to improve not only the direct oncolytic effect, but also the immune-stimulatory effect. The ability of these viruses to spread directly from cell to cell through fusion minimizes the release of viral progeny into the surrounding healthy tissue and systemic circulation, which we believe to be a key benefit in reducing off-target effects and avoiding viremia. This strategy could also function to minimize the counterproductive effects of neutralizing antibodies, as the virions are essentially hidden from inactivation due to their intracellular spread. Furthermore, because a single fusogenic virion can potentially lead to the incorporation of hundreds of neighboring cells into the growing syncytium, the virus has the ability to efficiently destroy tumors without the need for high titers of virus production within the tumor. This concept is supported by in vitro data indicating that, despite the fact that adenovirus-mediated GALV expression has a substantially inhibitory effect on virus replication at low multiplicities of infection (MOIs), a similar level of cytotoxicity can still be achieved compared to that of the control Ad vector [112].

Despite the accumulating data supporting the advantages of oncolytic viruses capable of inducing cell–cell fusion, it is expected that rationally designed combination therapies will offer the best chance of providing long-term survival benefits and harnessing the full potential of the host’s antitumor immune response. Particularly, immune checkpoint inhibitors revolutionized cancer therapy for certain tumor entities, but their efficacy depends on an immunologically active tumor microenvironment, and many solid tumors have proven difficult to treat by this approach [131]. Furthermore, these therapeutics are associated with severe side effects, and it is thought that the addition of synergistic agents in combination therapies can sufficiently activate the immune system, and potentially lower the dose of inhibitors needed in order to reduce toxicity. We and others rationalize that oncolytic viruses can represent optimal combinatorial agents to alter the tumor microenvironment and sensitize the tumor for subsequent immune checkpoint inhibition therapy. Specifically, with the induction of a strong immunogenic cell death through fusion, those oncolytic viruses expressing fusogenic proteins may provide the optimal setting for immune checkpoint inhibitors to ablate the immune tolerance in the tumor, and allow for optimal cytotoxic T-cell responses. This could potentially result in synergistic responses, and not only improve the outcome of treatment, but also reduce side effects, if a lower dose of checkpoint inhibitors could be used [132].

Despite the many potential benefits of fusogenic viruses as oncolytic agents, a valid safety concern should be considered, in that these viruses could potentially cause fusion of tumor cells with surrounding healthy cells, since the fusion process is not tumor specific. However, these viruses were applied in a multitude of clinical trials over several decades, and to our knowledge, there are no reports of fusion occurring within healthy tissue. Although it is not completely clear why this is the case, we believe that it is, at least in part, due to the capsule surrounding the tumor, which creates a physical barrier, preventing direct contact of the tumor cells with the surrounding tissue. Preclinical in vivo investigations on the tumor specificity of the measles virus was limited by the fact that it only enters human cells, meaning that, for the most part, xenograft mouse models are employed. These studies are naturally biased in the sense that virus replication is artificially restricted to the tumor, due to the lack of virus receptors on the mouse cells. This limitation was partially overcome with the generation of human CD46 transgenic mice [94,133]. In contrast, the Newcastle disease virus, which can infect a wide variety of host cells, was tested in numerous syngeneic animal models [85,134,135].

As we are currently witnessing major advancements in the field of viroimmunotherapeutics, and it is expected that increasing numbers of OV platforms will reach mainstream clinical application for cancer patients in the near future, this is indeed an exciting time to be working in the field of oncolytic virus development. Whether these vectors will be sufficiently effective in cancer destruction on their own, or whether they will be implemented as part of combination therapies, remains to be seen. Nevertheless, we predict that the development of fusogenic vectors for improved spread, potent immune stimulation, and enhanced safety will become a valuable strategy in the advancement of OV vectors for clinical translation.

## Figures and Tables

**Table 1:** Fusion proteins, their origin, and the new backbone viral vector, as well as a short summary of the effects in vitro and in vivo, are listed here.

Fusogenic Proteins	Viral Origin *Family*	Recipient Viral Backbone	Engineering and Effect	Literature
**FAST protein**	Reovirus *Reoviridae*	VSV	VSVΔM51 + p14 FAST protein → Activity against breast cancer spheroids → Survival prolongation in a primary 4T1 and a CT26 metastatic colon cancer model → Increased numbers of activated splenic cluster of differentiation 4 (CD4), CD8 cells in tumors	Le Boeuf et al., 2017 [104]
**GALV.fus**	GALV *Retroviridae*	HSV	HSV-1 + truncated GALV.fus → Enhanced antitumor effect and safety under a strict late viral promoter	Fu et al., 2003 [105]
			HSV-1 (Synco-2D) + GALV.fus → Double fusogenic due to using a syncytial HSV mutant after random mutagenesis → Destruction of a non-immunogenic murine mammary primary tumor and metastases through strong antitumor immunity provided by CD8^+^ T cells	Nakamori et al., 2004 [106]
			HSV-1 + GALV + Fcy::Fur (OncoVEX^GALV/CD^) → Increased tumor cell killing in vitro and tumor shrinkage (5–10 fold) in vivo → Tested in human squamous cell carcinoma and fibrosarcoma [107], head and neck squamous cell carcinoma [108], gastroesophageal cancer cell lines [109], superficial bladder cancer [110]	Simpson et al., 2006 [107] Price et al., 2010 [108] Wong et al., 2010 [109] Simpson et al., 2012 [110]
		AdV	AdV + GALV attached to a blocking ligand via a MMP-cleavable linker (AdM40) → Tumor regression and prolongation of survival in a U87 glioma model	Allen et al., 2004 [111]
		AdV	AdV5 + GALV.fus (ICOVIR16) under major late promoter → Enhanced tumor cell killing in a variety of tumor cell types (glioma), as well as enhanced spreading of the virus throughout melanoma or pancreatic tumors in vivo	Guedan et al., 2008 [112] Guedan et al., 2012 [38]
		Lentivirus	HIV-based self-inactivating vector with a transcriptionally disabled 3′ LTR + GALV → Eradication of established and actively growing human tumor xenografts	Diaz et al., 2000 [113]
**HIV envelope**	HIV *Retroviridae*	AdV	AdV5 + HIVenv → Increased dispersion within the cytoplasm as well as more efficient release of viral progeny	Li et al., 2001 [100]
**MV-F**	MV *Paramyxo-viridae*	VSV	VSVΔG + MV-F/HN → Higher viral yield, faster replication kinetics and larger fusogenic capabilities	Ayala-Breton et al., 2014 [101]
**NDV-F**	NDV *Paramyxo-viridae*	VSV	VSV + NDV-F (L289A) → Enhanced cytotoxic effects in vitro and a survival advantage over a non-fusogenic control virus in vivo	Ebert et al., 2004 [103]
**SV5-F**	SV5 *Paramyxo-viridae*	AdV	AdV5 + SV5-F → Potent bystander effect (Ratio of fusion (F)-transduced to non-transduced cells 1:100)	Gómez-Treviño et al., 2003 [114]

FAST: Fusion-Associated Small Trans-membrane; VSV: Vesicular Stomatitis Virus; GALV: Gibbon Ape Leukemia Virus; HSV: Herpes Simplex Virus; AdV: Adenovirus; AdV: Adenovirus; HIV: Human Immunodeficiency Virus; MV: Measles Virus; NDV: Newcastle Disease Virus; SV: Simian Parainfluenza Virus; MMP: matrix metalloproteinase; LTR: long terminal repeat.
